# Education, training, and accreditation of Neonatologist Performed Echocardiography in Europe—framework for practice

**DOI:** 10.1038/s41390-018-0078-9

**Published:** 2018-08-02

**Authors:** Yogen Singh, Charles Christoph Roehr, Cecile Tissot, Sheryle Rogerson, Samir Gupta, Kajsa Bohlin, Morten Breindahl, Afif El-Khuffash, Willem de Boode, on behalf of the European Special Interest Group “Neonatologist Performed Echocardiography” (NPE), WP de Boode, T Austin, K Bohlin, MC Bravo, CR Breatnach, M Breindahl, E Dempsey, A El-Khuffash, AM Groves, S Gupta, B Horsberg Eriksen, PT Levy, PJ McNamara, Z Molnar, E Nestaas, SR Rogerson, CC Roehr, M Savoia, U Schubert, CE Schwarz, A Sehgal, Y Singh, MG Slieker, C Tissot, R van der Lee, D van Laere, B van Overmeire, van Wyk

**Affiliations:** 1 Department of Neonatology and Paediatric Cardiology, Cambridge University Hospitals and School of Clinical Medicine University of Cambridge, Cambridge, UK; 2 University of Oxford, Department of Paediatrics, Newborn Services, John Radcliffe Hospital, Oxford University Hospitals, Headington, UK; 3 Department of Pediatrics, Clinique des Grangettes, Chêne Bougeries, Switzerland; 4 The Royal Women’s Hospital, Parkville, VIC Australia; 5 University Hospital of North Tees, Durham University, Stockton-on-Tees, UK; 6 Department of Neonatology, Karolinska University Hospital, Karolinska Institutet, Stockholm, Sweden; 7 Department of Neonatology, The Rotunda Hospital, Dublin, Ireland; 8 Department of Pediatrics, The Royal College of Surgeons in Ireland, Dublin, Ireland; 9 Department of Neonatology, Radboud University Medical Center, Radboud Institute for Health Sciences, Amalia Children’s Hospital, Nijmegen, The Netherlands; 10 Department of Neonatology, Rosie Hospital, Cambridge University Hospitals NHS Foundation Trust, Cambridge, UK; 11 Department of Neonatology, La Paz University Hospital, Madrid, Spain; 12 Karolinska University Hospital, Karolinska Institutet, Stockholm, Sweden; 13 INFANT Centre, Cork University Maternity Hospital, University College Cork, Cork, Ireland; 14 Division of Newborn Medicine, Mount Sinai Kravis Children’s Hospital, New York, NY USA; 15 University Hospital of North Tees, Durham University, Stockton-on-Tees, UK; 16 Department of Pediatrics, Møre and Romsdal Hospital Trust, Ålesund, Norway; 17 Department of Pediatrics, Washington University School of Medicine, Saint Louis, MO USA; 18 Department of Pediatrics, Goryeb Children’s Hospital, Morristown, NJ USA; 19 Departments of Pediatrics and Physiology, University of Toronto, Toronto, Canada; 20 John Radcliffe Hospital, Oxford, UK; 21 Institute of Clinical Medicine, Faculty of Medicine, University of Oslo, Oslo, Norway; 22 Department of Cardiology and Center for Cardiological Innovation, Oslo University Hospital, Rikshospitalet, Oslo, Norway; 23 Department of Paediatrics, Vestfold Hospital Trust, Tønsberg, Norway; 24 Department of Paediatrics, University of Oxford, John Radcliffe Hospital, Oxford, UK; 25 Azienda Ospedaliero-Universitaria S Maria della Misericordia, Udine, Italy; 26 Department of Clinical Science, Intervention and Technology, Karolinska Institutet, Stockholm, Sweden; 27 Department of Neonatology, University Children’s Hospital of Tübingen, Tübingen, Germany; 28 Department of Pediatrics, Monash University, Melbourne, VIC, Australia; 29 Addenbrooke’s Hospital, Cambridge University Hospitals NHS Foundation Trust, Cambridge, UK; 30 Department of Paediatric Cardiology, Radboudumc Amalia Children’s Hospital, Nijmegen, The Netherlands; 31 Department of Pediatrics, Antwerp University Hospital UZA, Edegem, Belgium; 32 Department of Neonatology, University Hospital Brussels, Brussels, Belgium; 33 L. Department of Paediatrics & Child Health, University of Stellenbosch, Cape Town, South Africa

## Abstract

There is a growing interest worldwide in using echocardiography in the neonatal unit to act as a complement to the clinical assessment of the hemodynamic status of premature and term infants. However, there is a wide variation in how this tool is implemented across many jurisdictions, the level of expertise, including the oversight of this practice. Over the last 5 years, three major expert consensus statements have been published to provide guidance to neonatologists performing echocardiography, with all recommending a structured training program and clinical governance system for quality assurance. Neonatal practice in Europe is very heterogeneous and the proximity of neonatal units to pediatric cardiology centers varies significantly. Currently, there is no overarching governance structure for training and accreditation in Europe. In this paper, we provide a brief description of the current training recommendations across several jurisdictions including Europe, North America, and Australia and describe the steps required to achieve a sustainable governance structure with the responsibility to provide accreditation to neonatologist performed echocardiography in Europe.

## Preamble

There is growing interest in the utility of echocardiography performed by neonatologists in clinical decision making for preterm and term infants with hemodynamic instability.^1–3^ In fact, recent studies demonstrate that Neonatologist-Performed Echocardiography (NPE) is already being used in many neonatal units across Europe.^4^ However, the clinical application of NPE, its availability, the expertise of personnel performing the studies, and access to pediatric cardiology support remain highly variable. This could relate to the heterogeneity of neonatology services in Europe^4^ and, more importantly, variable proximity between pediatric cardiology services and neonatal intensive care units (NICUs).

Despite its rapidly growing popularity and fast adoption into the clinical practice, there is a lack of a training program and accreditation process that can provide quality assurance to the clinicians.^5^ There are significant concerns among neonatologists and pediatric cardiologists alike regarding the level of expertise of end users and available training programs for emerging neonatologists. Misdiagnosis, resulting in inappropriate management, could potentially lead to patient harm.^6^ However, several studies have shown that the use of NPE in the neonatal intensive care unit has resulted in an improvement in patient care without any adverse effects.^7–9^ Over the last 5 years, three expert consensus statements have been published to provide training recommendations; all statements have emphasized the urgent need to develop a structured training program and standard operating procedures for clinical practice to provide quality assurance.^10–12^

The practice guidelines and recommendations for the use of targeted neonatal echocardiography (TNE), endorsed by The American Society of Echocardiography, European Association of Echocardiography, and Association for European Pediatric Cardiology, provide an excellent structured approach to standardized training and maintenance of competency.^11^ Certain aspects of the suggested outline for training are currently not achievable in many European countries.^6,12–14^ This has prompted the need to develop local training programs to account the diversity and uniqueness of training centers across Europe.

In 2016, an expert consensus statement on NPE training and accreditation in the UK was published. The contents have been endorsed by the British Congenital Cardiac Association, Pediatricians with Expertise in Cardiology Special Interest Group and Neonatologists with Interest in Cardiology and Haemodynamics group.^12^ More recently, the European Special Interest Group for NPE, endorsed by the European Society for Pediatric Research (ESPR) and the European Society for Neonatology (ESN), published its recommendations for NPE in Europe after considering the heterogeneous nature of training facilities, personnel, and infrastructure across Europe.^6^ They acknowledged, however, that to maintain the high standards of care and patient safety there should be minimum agreed standards for training and maintenance of NPE skills. A close collaboration with the pediatric cardiologists during the training and beyond is essential.

In Australia, a Neonatal Certificate in Clinician Performed Ultrasound (CCPU) has predated all the recently published guidelines and currently provides the only accredited training pathway in existence for cardiac, brain, and abdominal ultrasound in neonatology. The Australian Society of Ultrasound in Medicine (ASUM) should be commended for developing and instituting this program (http://www.asum.com.au). This program operates on the premise that CHD has been excluded by a pediatric cardiologist or an equivalently trained person, which is generally done by review of the initial scan by the nominated expert.

An overview of all current guidelines is summarized in Table 1.

**Table 1:** Current training guidelines

Responsible organization and nomenclature	ESPR NPE Guideline Neonatologist-performed echocardiography (NPE)	ASE/EAE/AEPC TNE Guideline Targeted neonatal echocardiography (TNE)	NoPE UK Guideline Neonatologist-performed echocardiography (NoPE)	CCPU Australian Guideline Clinician-performed ultrasound (CPU)
**Jurisdiction**	**Europe**	**North America**	**United Kingdom**	**Australia**
Pre-course preparation	Demonstrate understanding of the physics of ultrasound	Not specified	Basics of neonatal echo; attend echo course	Echo basic course; online physics of ultrasound course
**Phase 1**	**Basic echo training**	**Core/basic training**	**One phase of training**	**Basic grade**
Number of scans	>100 scans—total number determined by trainer	150 performed/150 reviewed	Minimum 100 scans	50 cardiac scans
Nature of scans	70% must be normal	80% of scans must be abnormal	50 with structural/functional pathology	Normal anatomy. NICU setting scans; PDA; PPHN
Echo competencies	Standard views; PW & CW Doppler; M-mode; Chamber dimensions; FS & EF; confirm normal structural anatomy	Standard views; PW & CW Doppler; M-Mode; Chamber dimensions; FS & EF; Note rule out CHD by cardiologist	Exposure to all commonly encountered disease states and recognition of abnormal anatomy	2D acquisition; routine views; FS; PW & CW Doppler; M-mode
Duration	6 months minimum	4–6 Months	12 months	Unspecified
Place of training	NICU and/or pediatric cardiology	Echo laboratory	Pediatric cardiology and NICU	NICU
Evaluation	5 observed echocardiograms	Formal evaluation unspecified	10 DOPS	Supervisor signoff
**Phase 2**	**Advanced echo training**	**Advanced training**		**Advance grade**
Number of scans	>100 scans—total number determined by trainer	150 performed/150 reviewed		Two-day advanced course and 50 cardiac ultrasounds
Nature of scans	Up to 20 scans with CHD	Unspecified—NICU setting		50% with abnormalities
Additional competencies	Interpret scans in the clinical context. Assessment of PDA; PPHN; myocardial performance; central lines	Determine systolic and diastolic function in context of loading conditions; pericardial fluid assessment; pulmonary hypertension; central line appraisal		PDA assessment; cardiac output measurements; PPHN assessment; recognize common CHD
Duration	6–12 months	4–6 months		Unspecified
Place of training	NICU and/or pediatric cardiology	NICU		NICU
Evaluation	Demonstrate normal anatomy; PDA; PPHN; infant with HIE	Direct supervision—unspecified		Supervisor signoff
Maintenance of competence	50 scans per annum; establish formal link with pediatric cardiology	100 scans per annum; participation in echo conferences and training courses	50 scans per annum; establish formal link with pediatric cardiology	Recertification every 5 years.

See text for further details

*NICU* neonatal intensive care unit, *PDA* patent ductus arteriosus, *PPHN* persistent pulmonary hypertension of the newborn, *PW* pulsed wave, *CW* continuous wave, *FS* fractional shortening, *EF* ejection fraction, *CHD* congenital heart disease, *HIE* hypoxemic ischemic encephalopathy

## Toward certification and accreditation in NPE across Europe

There is a lack of an overarching governance structure for NPE across Europe. The recently published training guidelines provide recommendations for training and skill acquisition but do not outline how those recommendations can be implemented across Europe. The aim is to form a pan European Neonatologist Performed Echocardiography Certification Program, which is endorsed by all the relevant stakeholders comprising a collaboration between neonatal and pediatric cardiology bodies. This program should be endorsed by the ESPR and the European Board of Neonatology (EBN) and its first role should be to provide accreditation to units capable of delivering the training requirements recently outlined by our group.^6^ Currently, there are two certification processes that neonatologists can avail of.

1. Certification and accreditation in CHD by the European Association of Cardiovascular Imaging (EACVI) and Association for European Pediatric Cardiology (AEPC): as the name suggests, this certification focusses on the identification and diagnosis of structural abnormalities (http://www.escardio.org). The European NPE working group acknowledges that this is a robust certification process, however, this accreditation is not the best option for NPE training, which is primarily focused on the performance of echocardiography to evaluate heart function and hemodynamic. The goal of NPE training is not to achieve competency in diagnosing complex congenital heart defects, pre- and postoperatively. Neonatologists trained in NPE should be confident in defining normality and recognizing abnormality on echocardiography. The needs of clinicians performing NPE are very different from those of pediatricians with expertise in cardiology or pediatric cardiologists.
2. In Australia, a Neonatal Certificate in Clinician Performed Ultrasound provides accreditation in neonatal cardiac, brain, and abdominal ultrasound, and is the only accredited training pathway currently in existence for neonatologists (http://www.asum.com.au). As this program does not require establishing structural normality of heart and includes other organ scans, it cannot be adopted per se toward a European standard. The governance structure of this program does not include a collaboration with pediatric cardiology.^6^

Our goal is to develop a pan European Neonatologist Performed Echocardiography Certification Program with close collaboration with the Association for European Pediatric Cardiology. The goal of certification is to set a European standard for competence and excellence in NPE, and ultimately protect patients from undergoing NPE examinations performed by unqualified persons. This would provide quality assurance to the neonatologists, pediatric cardiologists, parents and all other stake holders in providing care to the vulnerable babies in NICU. Our colleagues in Canada (some of whom are authors on this manuscript) have already started the process of achieving accreditation for targeted neonatal echocardiography (TNE) through the Royal College of Physicians of Canada via a 1 year of dedicated TNE training. This will be recognized as an area of a focused competency for which a diploma is awarded.

## The next steps

A recognized accreditation process will help in making the clinical governance robust and hence would provide quality assurance. Certification in NPE will bring credibility and professional legitimacy to clinicians performing NPE by demonstrating their competency in gaining this certification. We envisage the following key elements for NPE certification and accreditation process:

- Certification program should be run by the neonatologists with advanced echocardiography skills in collaboration with pediatric cardiologists under the auspices of European Society for Pediatric Research and European Board of Neonatology. We aim to get endorsement by the national working parties and interest groups in Neonatology and Pediatric Cardiology as well as the Association for European Pediatric Cardiology (AEPC).
- Certification program should involve a combination of summative assessment (NPE based exam) and formative assessment (structured feedback from DOPS, review of logbooks, and NPE trainer’s report). Re-certification process should involve review of the logbooks to maintain the competencies.
- Accreditation of NPE training centers across Europe and individuals carrying out the training; certification program should allow the assessment of local training infrastructure and should be accountable for the provision of accreditation, training oversight, and direct continuing assessment.
- Accreditation could be reviewed every 5 years for re-certification and be based on evidence of continuing professional development in the field, performance of a minimum number of NPE studies/year, review of the logbook of scans performed, and a potentially formal evaluation of image acquisition.

## Conclusion

In the absence of a standardized structured training program for NPE, there remains significant variation in skills sets of clinicians performing echocardiograms. The clinical governance structure for NPE remains sub-optimal and it is necessary to establish a quality assurance system that can ensure ongoing improvements in patient safety in NICU. There is an urgent need to develop standard operating procedures for clinical NPE programs, a comprehensive curriculum for training, and formalized mechanism of accreditation of centers in collaboration with pediatric cardiology. Furthermore, NPE should be incorporated into a revised version of the European Curriculum and Assessment Framework for Subspecialty Training in Neonatology. The European Society for Pediatric Research and European Board of Neonatology acknowledge the importance of streamlining the NPE training and are supporting the NPE working group in developing NPE accreditation/certification program that can be used across Europe and beyond.

## Appendix

**European Special Interest Group “Neonatologist-Performed Echocardiography” (NPE), endorsed by the European Society for Paediatric Research (ESPR) and the European Board of Neonatology (EBN)**

de Boode WP (chairman), Department of Neonatology, Radboud University Medical Center, Radboud Institute for Health Sciences, Amalia Children’s Hospital, Nijmegen, The Netherlands (willem.deboode@radboudumc.nl);

Austin T, Department of Neonatology, Rosie Hospital, Cambridge University Hospitals NHS Foundation Trust, Cambridge, UK (topun.austin@addenbrookes.nhs.uk)

Bohlin K, Department of Neonatology, Karolinska University Hospital, Karolinska Institutet, Stockholm, Sweden (kajsa.bohlin@ki.se)

Bravo MC, Department of Neonatology, La Paz University Hospital, Madrid, Spain (mcarmen.bravo@salud.madrid.org)

Breatnach CR, Department of Neonatology, The Rotunda Hospital, Dublin, Ireland (colm.breatnach@gmail.com)

Breindahl M, Karolinska University Hospital, Karolinska Institutet, Stockholm, Sweden (morten.breindahl@sll.se)

Dempsey E, INFANT Centre, Cork University Maternity Hospital, University College Cork, Ireland (g.dempsey@ucc.ie)

El-Khuffash A, Department of Neonatology, The Rotunda Hospital, Dublin, Ireland; Department of Pediatrics, The Royal College of Surgeons in Ireland, Dublin, Ireland (afifelkhuffash@rcsi.ie)

Groves AM, Division of Newborn Medicine, Mount Sinai Kravis Children’s Hospital, New York, NY, USA. (alan.groves@mssm.edu)

Gupta S, University Hospital of North Tees, Durham University, Stockton-on-Tees, UK (samir.gupta@nth.nhs.uk)

Horsberg Eriksen B, Department of Pediatrics, Møre and Romsdal Hospital Trust, Ålesund, Norway (beate.eriksen@me.com)

Levy PT, Department of Pediatrics, Washington University School of Medicine, Saint Louis, MO, USA; Department of Pediatrics, Goryeb Children’s Hospital, Morristown, NJ, USA (Levy_P@kids.wustl.edu)

McNamara PJ, Departments of Pediatrics and Physiology, University of Toronto, Toronto, ON, Canada (patrick.mcnamara@sickkids.ca)

Molnar Z, John Radcliffe Hospital, Oxford, UK (zoltan.Molnar@ouh.nhs.uk)

Nestaas E, Institute of Clinical Medicine, Faculty of Medicine, University of Oslo, Oslo, Norway; Department of Cardiology and Center for Cardiological Innovation, Oslo University Hospital, Rikshospitalet, Oslo, Norway; Department of Paediatrics, Vestfold Hospital Trust, Tønsberg, Norway (nestaas@hotmail.com)

Rogerson SR, The Royal Women’s Hospital, Parkville, VIC, Australia (sheryle.Rogerson@thewomens.org.au)

Roehr CC, Department of Paediatrics, University of Oxford, John Radcliffe Hospital, Oxford, UK (charles.roehr@paediatrics.ox.ac.uk)

Savoia M, Azienda Ospedaliero-Universitaria S. Maria della Misericordia, Udine, Italy (marilena.savoia@gmail.com)

Schubert U, Department of Clinical Science, Intervention and Technology, Karolinska Institutet, Stockholm, Sweden (ulfschubert@gmx.de)

Schwarz CE, Department of Neonatology, University Children’s Hospital of Tübingen, Tübingen, Germany (c.schwarz@med.uni-tuebingen.de)

Sehgal A, Department of Pediatrics, Monash University, Melbourne, VIC, Australia (arvind.sehgal@monash.edu)

Singh Y, Addenbrooke’s Hospital, Cambridge University Hospitals NHS Foundation Trust, Cambridge, UK (yogen.Singh@nhs.net)

Slieker MG, Department of Paediatric Cardiology, Radboudumc Amalia Children’s Hospital, Nijmegen, The Netherlands (Martijn.Slieker@radboudumc.nl)

Tissot C, Department of Pediatrics, Clinique des Grangettes, Chêne Bougeries, Switzerland (cecile.tissot@hotmail.com)

van der Lee R, Department of Neonatology, Radboud University Medical Center, Radboud Institute for Health Sciences, Amalia Children’s Hospital, Nijmegen, The Netherlands (Robin.vanderLee@radboudumc.nl)

van Laere D, Department of Pediatrics, Antwerp University Hospital UZA, Edegem, Belgium (david.VanLaere@uza.be)

van Overmeire B, Department of Neonatology, University Hospital Brussels, Brussels, Belgium (bart.van.overmeire@erasme.ulb.ac.be)

van Wyk, L.Department of Paediatrics and Child Health, University of Stellenbosch, Cape Town, South Africa (lizelle@sun.ac.za)
